# Bioactivity of *Matricaria chamomilla*, *Echinacea purpurea*, *Thymbra capitata* and *Ocimum basilicum* Hydrolates and Essential Oils in View of Their Application in the Skin

**DOI:** 10.3390/molecules31071156

**Published:** 2026-03-31

**Authors:** João Vasco Valente, Carolina Proença Gomes, Ana Sofia Oliveira, Joana Rolo, Carlos Gaspar, Débora Caramelo, José Carlos Gonçalves, Fernanda Delgado, Luiza Breitenfeld, Ana Paula Duarte, Rita Palmeira-de-Oliveira, José Martinez-de-Oliveira, Ana Palmeira-de-Oliveira

**Affiliations:** 1 RISE-Health, Faculty of Health Sciences, University of Beira Interior, 6200-209 Covilhã, Portugal; joaovascov@gmail.com (J.V.V.); carolinagomes1998@hotmail.com (C.P.G.); ana_g2s@hotmail.com (A.S.O.); joanarolo@fcsaude.ubi.pt (J.R.); cgaspar@fcsaude.ubi.pt (C.G.); luiza@fcsaude.ubi.pt (L.B.); apduarte@fcsaude.ubi.pt (A.P.D.); rpo@fcsaude.ubi.pt (R.P.-d.-O.); jmo@fcsaude.ubi.pt (J.M.-d.-O.); 2 NuESA-Health and Environment Study Unit, Faculty of Health Sciences, University of Beira Interior, 6200-209 Covilhã, Portugal; 3 Faculty of Health Sciences, University of Beira Interior, 6200-209 Covilhã, Portugal; 4 Labfit-HPRD: Health Products Research and Development Lda, 6200-209 Covilhã, Portugal; 5 School of Agriculture, Polytechnic Institute of Castelo Branco, 6000-909 Castelo Branco, Portugal; dbrcaramelo@gmail.com (D.C.); jcgoncalves@ipcb.pt (J.C.G.); fdelgado@ipcb.pt (F.D.); 6 CBPBI: Biotech Plant Lab of Beira Interior, 6000-909 Castelo Branco, Portugal; 7 CERNAS: Research Center for Natural Resources, Environment and Society, Agriculture Science Research Group, 6000-909 Castelo Branco, Portugal

**Keywords:** aromatic plants, bioactive compounds, microbial growth, cytotoxicity, free radicals

## Abstract

The numerous health benefits associated with the use of plants in traditional medicine can be linked to secondary metabolites in the products obtained through the hydrodistillation process, such as hydrolates and essential oils (EOs). This study aimed to assess the chemical profile and the antioxidant, antimicrobial, cytotoxic, and anti-inflammatory activities of two hydrodistillation products obtained from four plants belonging to two distinct families: chamomile (*Matricaria chamomilla*), purple coneflower (*Echinacea purpurea*), conehead thyme (*Thymbra capitata*), and basil (*Ocimum basilicum*). The EOs were found to be more effective than hydrolates in inhibiting microorganisms’ growth, registering MIC values equal to or higher than 50% (*v*/*v*), except for *T. capitata*. Only *T. capitata* demonstrated the ability to reduce NO levels with both hydrolate and EO. Moreover, it inhibited the cell growth of RAW 264.7, 3T3, and HaCaT lines at the tested concentrations. In contrast, *O. basilicum* EO did not affect the cell proliferation of the tested cell lines in concentrations below 0.063% (*v*/*v*) and showed a significant reduction in the macrophage NO production at all concentrations. Thus, EOs showed a superior biological activity than hydrolates. In particular, *O. basilicum* EO was found to be a promising candidate for therapeutic applications on the skin.

## 1. Introduction

Over the years, plants have been shown to possess in their constitution, several phytochemicals with health-promoting effects [1]. By modulating specific metabolic pathways in the human body [2,3], these compounds have been linked to various biological properties, including antioxidant, antimicrobial, antiviral, and anticancer effects [4]. Plant secondary metabolites are naturally produced by plants to carry out various functions like protecting against UV rays, repelling pathogens and herbivores, and enhancing pollination [5]. Although they are directly related to fundamental physiological processes (e.g., growth and reproduction), they also contribute to enhancing plant health and survivability by protecting against biotic and abiotic stress [6,7,8]. These include a variety of bioactive compounds, such as alkaloids, terpenoids, and polyphenols, which already underlie a wide range of biological activities [9,10,11,12]. Alkaloids often exhibit antimicrobial effects by compromising bacterial cell membrane permeability, which destabilizes the cell and causes lysis [13], while terpenoids (e.g., monoterpenes and sesquiterpenes) provide defenses against oxidant and inflammatory agents by scavenging free radicals and modulating inflammatory pathways [14]. Indeed, hydrolates and EOs, which are obtained by hydrodistilling of some plant parts [15], exhibit those properties, owing to monoterpenes, sesquiterpenes, alcohols, aldehydes, ketones, and esters [16,17].

In modern society, access to healthcare and/or medical services is very limited. Indeed, in certain countries, it is estimated that approximately 80% of the population lacks access to healthcare products and generic medicine treatments [18]. Additionally, the insufficient therapeutic effect or the incidence of adverse effects led people to find alternative or complementary medicines [19,20]. As a result, traditional medicine derived from natural bioactive compounds has emerged as a viable alternative, demonstrating therapeutic potential against cardiovascular disorders, rheumatoid arthritis, osteoporosis, and skin diseases [21,22,23,24,25,26]. Furthermore, bioactive compounds have played a crucial role in the discovery and development of drugs, offering an abundant supply of novel chemicals owing to their biodiversity [27]. Unlike synthetic products, which are known to present certain instability and potential toxicity, natural products offer better biocompatibility and, therefore, are being prioritized as sustainable alternatives by various industries such as food processing, food supplement production, and pharmaceuticals [28,29,30,31]. Also, exploring new sources of biological agents derived from plants holds significant potential as an alternative for inhibiting the growth of certain resistant microorganisms. This approach could offer new strategies to address the currently faced challenges of antibiotic drug resistance, affordability, and efficacy [32,33]. Still, and despite their curative effects, certain plant-derived products may exhibit significant toxicological liabilities, including herb-induced kidney and liver failure, as well as cardiotoxic and carcinogenic activity [34,35,36]. Therefore, there is an ongoing need for additional safety information to facilitate the successful development and validation of new drugs and therapeutics derived from unexplored plants utilized in traditional pharmacopeias [37].

Skin, the body’s largest organ, serves as a protective barrier against external harmful agents, including microbes, chemicals and sunlight, and also plays a synergistic role in regulating temperature and body fluid balance [24]. It consist in a complex network of multiple cell types that communicate with each other in order to maintain various vital processes, such as inflammation, immune response, angiogenesis and wound healing [38]. Typically, wound infections are characterized by erythema, heat and purulent exudate and represent a major factor that leads to delayed wound healing and unhealthy granulation tissue breakdown [39]. Also, damages on the skin increases the risk of microorganism colonization and subsequent infection [40]. During the healing process, wounds can become infected by pathogenic bacteria, such as *Staphylococcus aureus*, *Pseudomonas aeruginosa*, *Streptococcus* spp., and *Escherichia coli* [40] as well as opportunistic fungi, including *Aspergillus* and *Candida* spp., which can cause severe human infections [41]. At the wound site, in the course of the inflammatory process, there is an increase in reactive oxygen species (ROS) levels [42]. In the absence of proper mediation, ROS can induce cell lesions, such as membrane disorganization and protein oxidation, thereby resulting in additional tissue injury and damage [42]. Hence, maintaining a balance between the production of ROS and antioxidants is crucial for effective tissue repair [42]. Moreover, ROS play a significant role in the process of premature skin aging by causing damage to the connective tissue in the dermis [43]. The interaction between ROS and lipids can also give rise to detrimental effects on the integrity of the epidermal barrier [44]. This leads to increased water loss and triggers the production of prostaglandins, ultimately resulting in skin dryness and inflammation of the epithelial layer [44]. Although the human body possesses natural mechanisms to counteract ROS, including enzymes (catalase and superoxidase dismutase), low molecular weight antioxidants (uric acid and glutathione), vitamins (ascorbic acid and α-tocopherol), and carotenoids, external sources of antioxidants are important to assist and prevent oxidative damage from ROS [45]. Some of them have been found in plants and revealed potential to act as an active part in delaying aging, reducing inflammation, and preventing some cancers [46,47]. Therefore, plant-based products emerge as strong candidates to reduce the accumulation of reactive oxygen species responsible for proteins, lipids, and DNA degradation, while protecting the wound from infection and enhancing the healing process [43,48].

Considering the above-mentioned properties and the growing interest in natural ingredients as sustainable and alternative source of biologically active compounds, plant species from the central Portugal belonging to two distinct families were selected: *Matricaria chamomilla* (L.), *Echinacea purpurea* (L.), *Thymbra capitata* (L.) (from Asteraceae family), and *Ocimum basilicum* (L.) (from Lamiaceae family). Thus, this study aims to assess the antioxidant, antimicrobial, cytotoxicity, and anti-inflammatory activity of hydrolates and essential oils obtained from the selected aromatic plants. Through comprehensive characterization of these hydrodistillation byproducts and products, the research seeks to enhance understanding of their biological effects and provide valuable insights to support their potential application in cosmetic and pharmaceutical applications.

## 2. Results

### 2.1. Chemical Characterization

The hydrolates and EO analyzed by GC-MS are shown in Table 1. The *M. chamomilla* hydrolate and EO are predominantly constituted by oxanes with very similar compounds, named bisabolol oxide A, with percentages (%) of 80.67 and 62.42, respectively. Despite the flavonoid trans-verbenol being the prevalent compound in E. purpurea hydrolate, it only registered a percentage of 11.70. The *E. purpurea* EO major compounds were the sesquiterpenes germacrene D and caryophyllene oxide, with 10.64 and 9.52%, respectively. As mentioned before, *T. capitata* and *O. basilicum* hydrodistillation products were already reported in a previous study of the group [49]. However, it is worth mentioning that once more, a very low yield was obtained for the *O. basilicum* EO, and therefore, its chemical characterization was not possible. In this study, it was found that carvacrol was the main compound of both *T. capitata* hydrolate and EO, and eugenol was the major component of *O. basilicum* hydrolate.

### 2.2. Antimicrobial Activity

The activity of the various plant extracts was evaluated against bacteria and fungi naturally present in the skin microbiota. Visual MIC values were obtained for hydrolates and EOs against Gram-positive bacteria *Staphylococcus aureus* and *Cutibacterium acnes*, Gram-negative *Escherichia coli* and *Pseudomonas aeruginosa*, and fungi *Aspergillus brasiliensis* and *Candida albicans* (Table 2). Overall, EOs were more effective in inhibiting microorganisms’ growth than hydrolates since the latter registered MIC values equal to or higher than 50%, except for *T. capitata*. Indeed, at a concentration of 12.5% (*v*/*v*), the *T. capitata* hydrolate impaired the growth of *P. aeruginosa*, *S. aureus*, *C. albicans*, and *A. brasiliensis*, and at 25% (*v*/*v*) inhibited the strains of *E. coli* and *C. acnes*. Regarding the EOs, the *T. capitata* showed the highest potential to inhibit microbial growth against the various strains, especially for *C. albicans* and *A. brasiliensis* (MIC = 0.0025%). Also, *C. acnes was* revealed to be the most susceptible microorganism with lower visual MIC values (0.03 and 0.006%) within the EOs.

### 2.3. Antioxidant Activity

The antioxidant potential of the hydrolates and EOs, evaluated through the DPPH radical scavenging assay, is shown in Table 3. Ascorbic acid is a well-known strong antioxidant that was used as a standard. All hydrolates registered high values of EC_50_ and, in turn, low IAA, which indicates a low antioxidant capacity. However, the *O. basilicum* EO was able to scavenge DPPH radicals, obtaining a low IC_50_ and high IAA, and therefore was classified as a strong antioxidant, while the *T. capitata* EO presented low antioxidant capacity.

### 2.4. Cytotoxicity Evaluation

**Figure 1:**
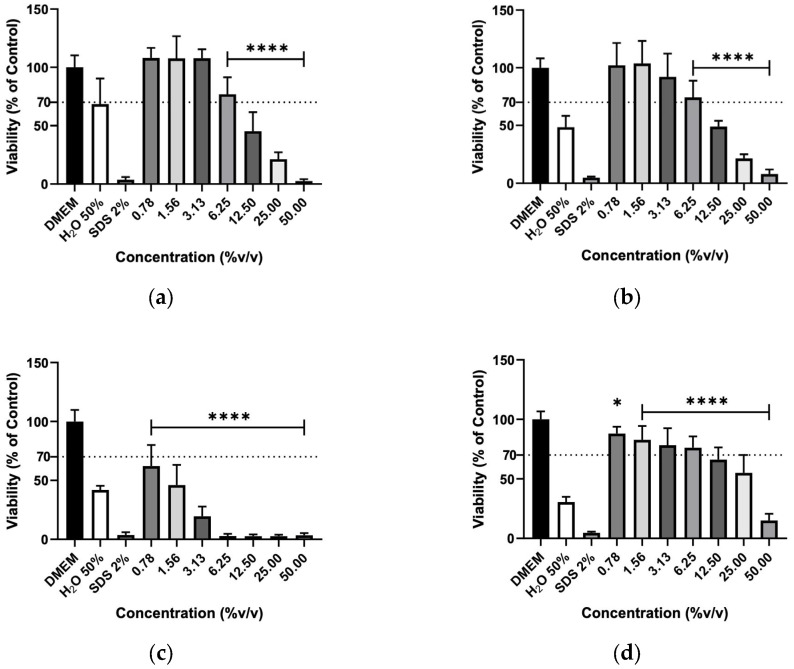
Cellular viability (%) of RAW 267.4 cells after exposure to (**a**) *M. chamomilla*, (**b**) *E. purpurea*, (**c**) *T. capitata*, and (**d**) *O. basilicum* hydrolates determined by the MTT assay. DMEM medium and SDS 2% solutions were used as negative and positive controls, respectively. H_2_O 50% solution was employed to mimic the dilution factor accomplished when testing the maximum concentration (50%) on hydrolates. Bars and lines indicate the mean ± SD of three independent assays and are represented as a percentage of the control. Significant differences when compared with control (DMEM) are represented as * (*p* < 0.05) and **** (*p* < 0.0001).

**Figure 2:**
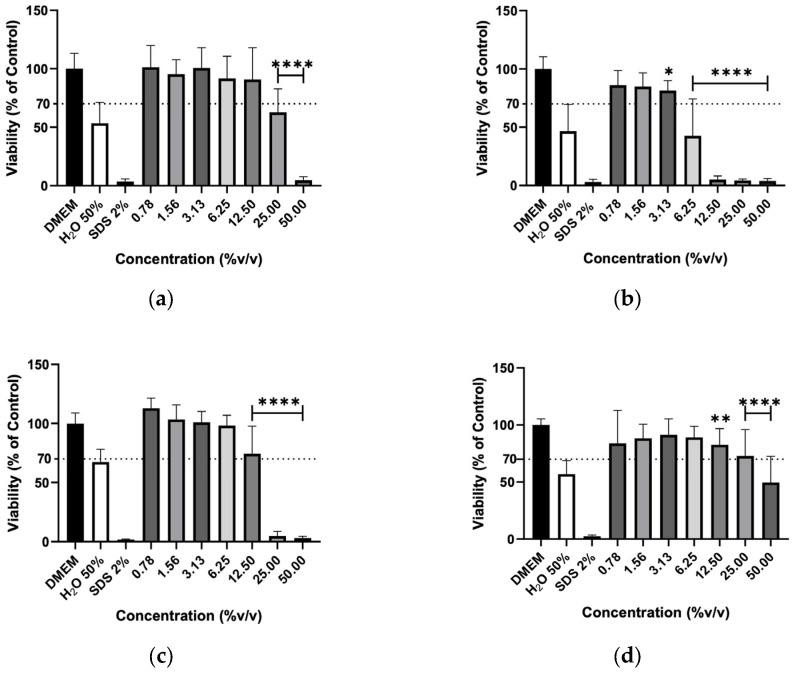
Cellular viability (%) of 3T3 cells after exposure to (**a**) *M. chamomilla*, (**b**) *E. purpurea*, (**c**) *T. capitata*, and (**d**) *O. basilicum* hydrolates determined by the MTT assay. DMEM medium and SDS 2% solutions were used as negative and positive controls, respectively. H_2_O 50% solution was employed to mimic the dilution factor accomplished when testing the maximum concentration (50%) on hydrolates. Bars and lines indicate the mean ± SD of three independent assays and are represented as a percentage of the control. Significant differences when compared with control (DMEM) are represented as * (*p* < 0.05), ** (*p* < 0.01) and **** (*p* < 0.0001).

**Figure 3:**
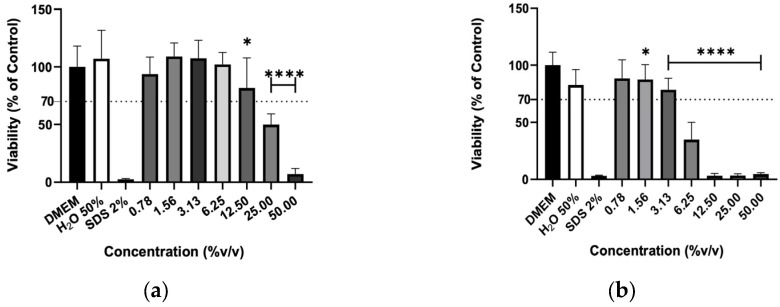

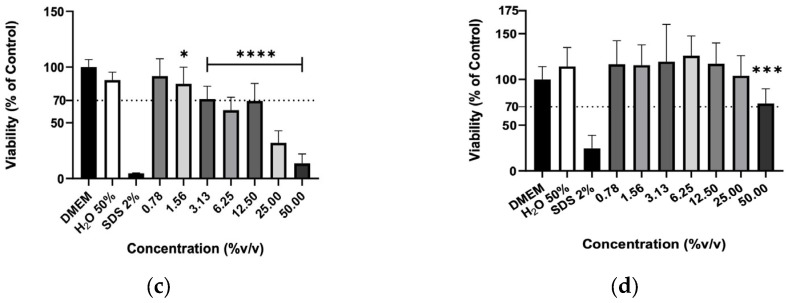
Cellular viability (%) of HaCaT cells after exposure to (**a**) *M. chamomilla*, (**b**) *E. purpurea*, (**c**) *T. capitata*, and (**d**) *O. basilicum* hydrolates determined by the MTT assay. DMEM medium and SDS 2% solutions were used as negative and positive controls, respectively. H_2_O 50% solution was employed to mimic the dilution factor accomplished when testing the maximum concentration (50%) on hydrolates. Bars and lines indicate the mean ± SD of three independent assays and are represented as a percentage of the control. Significant differences when compared with control (DMEM) are represented as * (*p* < 0.05), *** (*p* < 0.001) and **** (*p* < 0.0001).

The effect of hydrolates on the viability of murine macrophages (RAW 264.7), murine fibroblasts (3T3) and human keratinocytes (HaCaT) cells was evaluated to elucidate the biocompatibility of the plant extracts. The hydrolates were tested at the same concentrations as for antimicrobial activity; however, for EOs, the highest concentration tested was determined by ensuring that the solvent (DMSO) did not cause a decrease in cell viability greater than 30% Overall, the increase in hydrolate concentration decreased the RAW 267.4 cells’ viability in a dose-dependent manner, as illustrated in Figure 1. However, at low concentrations, such as 0.78 and 1.56%, the M. chamomilla and E. purpurea barely affected the cell’s viability. Regarding the 3T3 cells (Figure 2), the concentrations of 0.78, 1.56, 3.13 and 6.25% did not significantly reduce the cell viability of the hydrolates, except for the E. purpurea. Nevertheless, the same did not happen at the concentrations of 25 and 50%, which led to a significant reduction in the 3T3 cells’ viability. The HaCaT cells (Figure 3) showed more damage at higher concentrations. However, the E. purpurea and T. capitata hydrolates significantly reduced the cell’s viability at concentrations above 1.56% (*v*/*v*).

**Figure 4:**
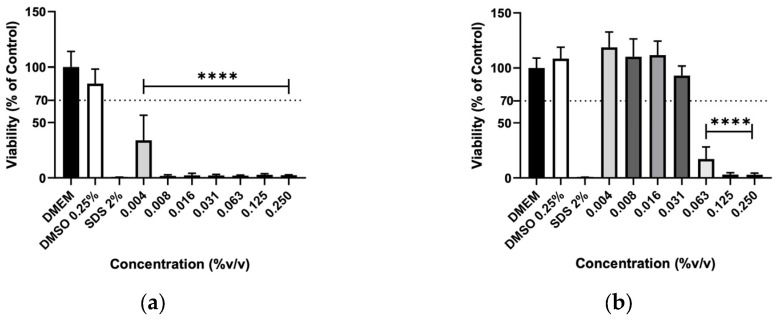
Cellular viability (%) of RAW 267.4 cells after exposure to (**a**) *T. capitata*, and (**b**) *O. basilicum* EOs determined by the MTT assay. DMEM medium and SDS 2% solutions were used as negative and positive controls, respectively. The DMSO 0.25% solution was tested at the maximum concentration utilized as a solvent to eliminate the possibility of interference in cellular toxicity. Bars and lines indicate the mean ± SD of three independent assays and are represented as a percentage of the control. Significant differences when compared with control (DMEM) are represented as **** (*p* < 0.0001).

**Figure 5:**
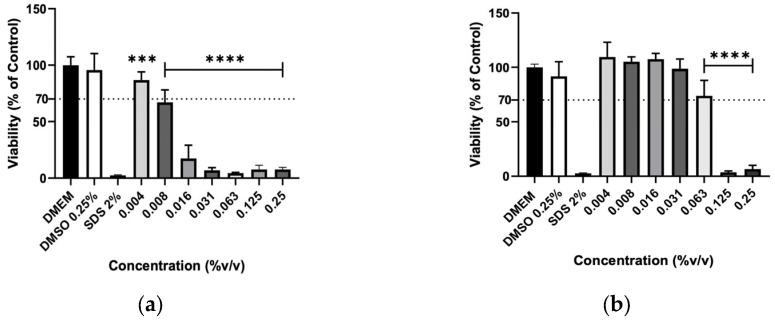
Cellular viability (%) of 3T3 cells after exposure to (**a**) *T. capitata*, and (**b**) *O. basilicum* EOs determined by the MTT assay. DMEM medium and SDS 2% solutions were used as negative and positive controls, respectively. The DMSO 0.25% solution was tested at the maximum concentration utilized as a solvent to eliminate the possibility of interference in cellular toxicity. Bars and lines indicate the mean ± SD of three independent assays and are represented as a percentage of the control. Significant differences when compared with control (DMEM) are represented as *** (*p* < 0.001) and **** (*p* < 0.0001).

**Figure 6:**
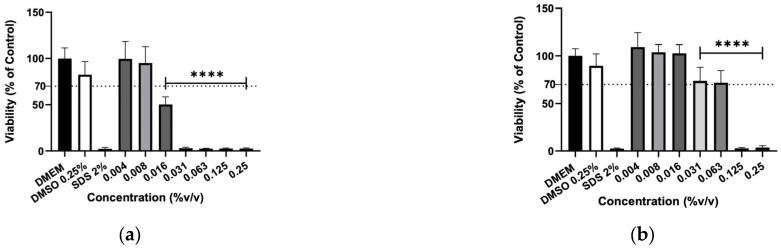
Cellular viability (%) of HaCaT cells after exposure to (**a**) *T. capitata*, and (**b**) *O. basilicum* EOs determined by the MTT assay. DMEM medium and SDS 2% solutions were used as negative and positive controls, respectively. The DMSO 0.25% solution was tested at the maximum concentration utilized as a solvent to eliminate the possibility of interference in cellular toxicity. Bars and lines indicate the mean ± SD of three independent assays and are represented as a percentage of the control. Significant differences when compared with control (DMEM) are represented as **** (*p* < 0.0001).

The effect of EOs was also evaluated on murine macrophages (RAW 264.7), murine fibroblasts (3T3) and human keratinocytes (HaCaT) cells to assess their biocompatibility. Due to very low yields obtained during the hydrodistillation process, *M. chamomilla* and *E. purpurea* EOs were not obtained. The results show the number of viable cells evaluated by MTT assay after the 24h exposure to the concentrations from 0.004 to 0.25% (*v*/*v*) (Figure 4, Figure 5 and Figure 6). The EOs decreased the cell viability of macrophages in a dose-dependent manner, as did the hydrolates. Also, higher concentrations, such as 0.063, 0.125, and 0.25%, significantly reduced the cell viability in the EOs tested. The *T. capitata* EO successfully inhibited the cells’ growth at every tested concentration on RAW 264.7 and 3T3 cells, whereas the same effect was registered on HaCaT cells for concentrations equal to or higher than 0.016%. Otherwise, the *O. basilicum* EO only showed significant reductions in the proliferation of the tested cell lines at high concentrations, such as 0.063, 0.125 and 0.250%.

### 2.5. Anti-Inflammatory Activity

#### 2.5.1. NO Production

**Figure 7:**
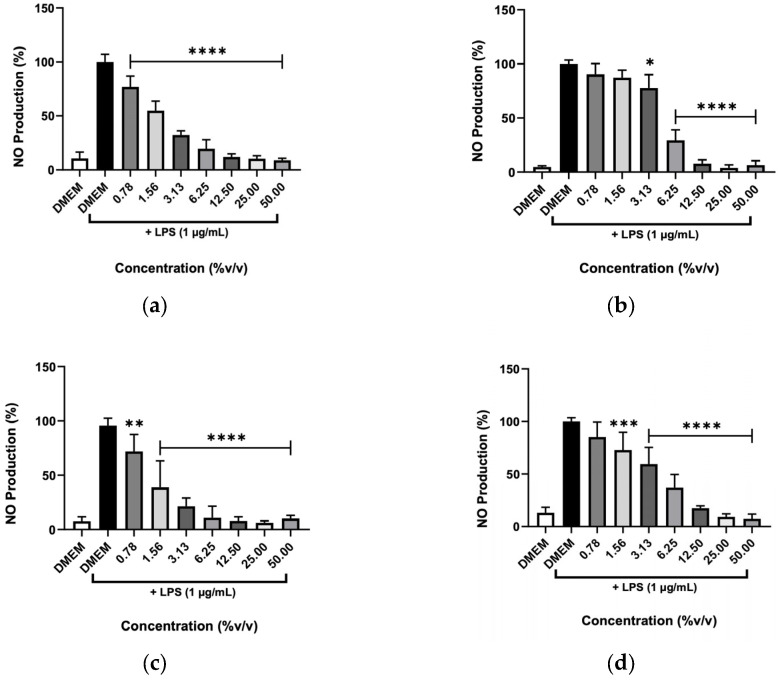
Effect of (**a**) *M. chamomilla*, (**b**) *E. purpurea*, (**c**) *T. capitata*, and (**d**) *O. basilicum* hydrolates in LPS-stimulated RAW 264.7 cells after 24h. The results show the levels of NO stable metabolites (nitrites) in the cell supernatants, as measured by the Griess assay. Negative control with only DMEM medium was included to address the basal NO production. Bars and lines indicate the mean ± SD of three independent assays and are represented as a percentage of control cells exposed to LPS (DMEM + LPS). Significant differences when compared with control (DMEM + LPS) are represented as * (*p* < 0.05), ** (*p* < 0.01), *** (*p* < 0.001) and **** *(p* < 0.0001).

**Figure 8:**
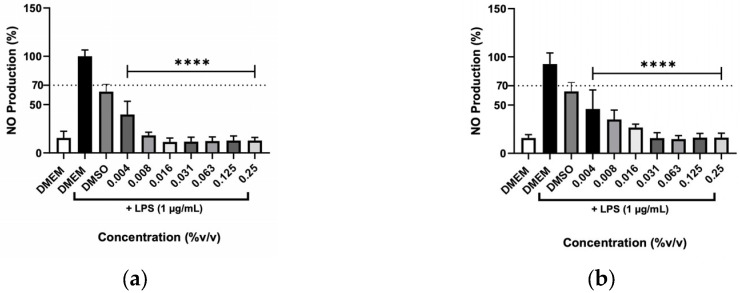
Effect of (**a**) *T. capitata*, and (**b**) *O. basilicum* EOs in LPS-stimulated RAW 264.7 cells after 24 h. The results show the levels of NO stable metabolites (nitrites) in the cell supernatants, as measured by the Griess assay. Negative control with only DMEM medium was included to address the basal NO production. Bars and lines indicate the mean ± SD of three independent assays and are represented as a percentage of control cells exposed to LPS (DMEM + LPS). Significant differences when compared with control (DMEM + LPS) are represented as **** (*p* < 0.0001).

The anti-inflammatory activity of the various hydrolates and EOs was evaluated through their ability to inhibit the NO production by macrophages after being stimulated with the endotoxin LPS (Figure 7 and Figure 8). This stimulus was used to produce a response in the cells that mimics a state of inflammation. Then, NO metabolites (nitrites) measured by the colorimetric Griess reaction were used as a parameter to determine the extract’s anti-inflammatory potential. Figure 7 shows that the hydrolates of M. chamomilla and T. capitata significantly reduced the NO levels in stimulated macrophages at all concentrations tested. For the remaining hydrolates, namely *E. purpurea* and *O. basilicum*, only low concentrations (0.78 and 1.56%) did not decrease the NO production. Regarding both EOs, shown in Figure 8, it can be observed that all concentrations significantly reduced the NO levels, which could indicate anti-inflammatory capacity.

#### 2.5.2. Scavenging Activity

**Figure 9:**
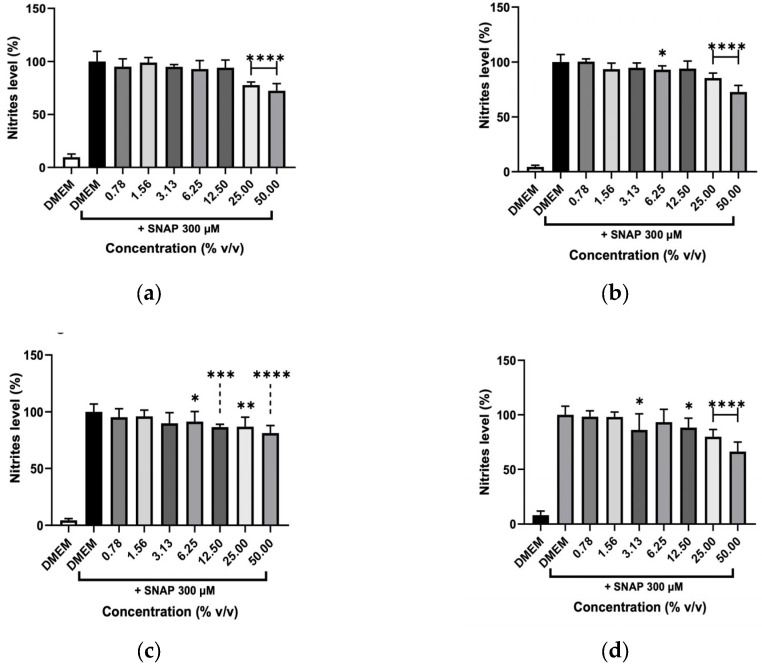
NO scavenging capacity of (**a**) *M. chamomilla*, (**b**) *E. purpurea*, (**c**) *T. capitata*, and (**d**) *O. basilicum* hydrolates triggered by the NO donor, SNAP. Negative control with just DMEM medium was incorporated to evaluate the basal nitrite values. Bars and lines indicate the mean ± SD of three independent experiments and are represented as a percentage of DMEM medium with SNAP (DMEM+SNAP). Significant differences when compared with control (DMEM+SNAP) are represented as * (*p* < 0.05), ** (*p* < 0.01), *** (*p* < 0.001) and **** (*p* < 0.0001).

**Figure 10:**
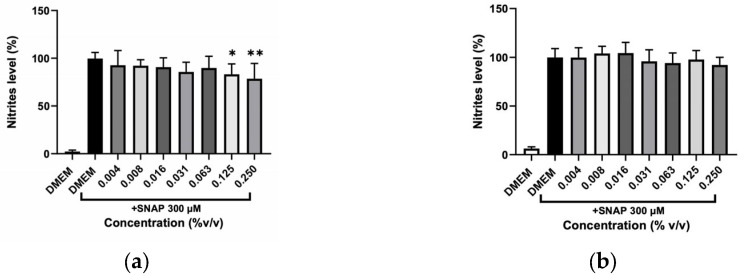
Effect of NO scavenging capacity of (**a**) T. capitata and (**b**) O. basilicum EOs triggered by the NO donor, SNAP. Negative control with just DMEM medium was incorporated to address the basal nitrite values. Bars and lines indicate the mean ± SD of three independent experiments and are represented as a percentage of DMEM medium with SNAP (DMEM+SNAP). Significant differences when compared with control (DMEM + SNAP) are represented as * (*p* < 0.05) and ** (*p* < 0.01).

A scavenging test was performed to clarify whether the decrease in NO was due to inhibition of the inflammatory process induced by LPS or whether hydrolates and EOs could remove NO from the cell’s medium. The reaction between a NO donor (SNAP) and the different plant preparations at various concentrations is illustrated in Figure 9 and Figure 10. The hydrolates (Figure 9), only at high concentrations (12.50, 25, and 50%) were able to significantly reduce the NO levels, which indicates that only these concentrations can scavenge NO from SNAP. In contrast, low concentrations of extracts almost did not affect the NO. The EOs in Figure 10 revealed that no effect was detected for *O. basilicum*; whereas the *T. capitata* exhibited the ability to directly scavenge NO in the cell’s medium when high concentrations (0.125 and 0.250%) of EO were tested.

## 3. Discussion

This study described the chemical composition alongside the biological activities of plant hydrolates and EOs. A thorough understanding of these effects is essential to substantiate their prospective use in cosmetic and pharmaceutical applications.

The *M. chamomilla* hydrolate and EO are primarily composed of sesquiterpenes, including bisabolol oxide A and bisabolol oxide B, which is in accordance with previous studies addressing this EO [50,51,52,53,54]. Other reports identified β-Farnesene (sesquiterpene) as the predominant compound of *M. chamomilla* EO (52.73 and 42.20%), with bisabolol oxide B, bisabolol, and bisabolol oxide A detected as subsequent major compounds [55,56,57]. Furthermore, EO from Moroccan-cultivated plants featured germacrene (19.46%) and α-curcumene (19.00%), both sesquiterpenes, as principal constituents. Collectively, these findings suggest that *M. chamomilla* EO is primarily composed of sesquiterpenes, though constituent profiles vary by cultivar and region. Regarding *E. purpurea* EO, a study conducted by Dosoky et al. [58] identified the sesquiterpene germacrene D as its major constituent (42.0 ± 4.61%) and, although found at a lower proportion in the present study, it was still the most abundant compound (10.64%). Despite being identified α-phellandrene (monoterpene) as the second most abundant compound of *E. purpurea* EO [58], the high sesquiterpenes content of this EO is corroborated by the presence of β-caryophyllene (5.75 ± 1.72%), γ-curcumene (5.03 ± 1.96%), and δ-cadinene (3.31 ± 0.61%) [58], as well as caryophyllene oxide (9.52%), which emerged as the second most abundant compound in the current work. Unfortunately, to the author’s best knowledge, there is no available data in the literature about the GC-MS analysis of *E. purpurea* hydrolate. Also, *O. basilicum* EO demonstrated very promising effects across different biological activities; however, consistent with a previously published paper from the group [49], its chemical profile could not be fully determined. This outcome suggests that the hydrodistillation process should be further optimized or an alternative extraction methodology applied, for example, supercritical CO_2_ should be considered [59].

The activity against skin microbiota microorganisms, which could be related to skin disorders, was assessed. In the case of *O. basilicum*, it was found that the EO has inhibited the growth of *E. coli* [60] and, actually, can sometimes have a bactericidal effect on that strain [61]. The EO that resulted from the distillation of *M. chamomilla* revealed antibacterial activity against *S. aureus* and *P. aeruginosa*, although it was only modest activity [56]. In another study, the same EO was capable of inhibiting the growth of *S. aureus*, *P. aeruginosa* and *E. coli*, being more evident on Gram-negative bacteria, such as *P. aeruginosa* (MIC = 0.04 ± 0.02 mg/L) and *E. coli* (MIC = 0.17 ± 0.07 mg/L) [50]. Moreover, the authors identified bisabolol oxide A as one of the principal compounds within the oil of *M. chamomilla* EO, suggesting a potential correlation between this compound and its antibacterial properties. However, in the present study, such an analysis was not feasible, as previously mentioned. Moukhles et al. [62] reported that both *T. capitata* hydrolate and EO possess remarkable antimicrobial activity against Gram-positive (*S. aureus*) and Gram-negative (*E. coli*) bacteria, reaching the same MIC for both strains (0.031% *v*/*v*). Interestingly, the present study shows that *T. capitata* was the most effective species in inhibiting the growth of the microorganisms. Since EOs are lipophilic compounds, they can cross the cell membrane, permeate into the intracellular medium, and disrupt structures of different layers of polysaccharides, phospholipids and fatty acids [16]. This may result in cytoplasm coagulation, leakage of macromolecules and lysis [63,64,65]. Indeed, terpenes’ lipophilic character have been associated with their antimicrobial activity [66]. Generally, terpenes are more likely to affect Gram-positive than Gram-negative bacteria [67]. However, monoterpenes already shown antibacterial activity against both Gram-positive (*S. aureus*) and Gram-negative (*E. coli*) bacteria [66,68]. Furthermore, the α-terpineol, a known monoterpene that was detected in the *E. purpurea* hydrolate, had inhibited the growth of penicillin-resistant bacterial strains [69]. In addition, Brehm-Stecher and Johnson [70] found that sesquiterpenes can improve the bactericidal effect of antibiotics by enhancing their permeability and susceptibility. Until now, terpenes have been reported as antimicrobial agents, which may be related to the number of carbons in the hydrophobic chain of the hydrophilic hydroxyl group [71]. In opposition, a more recent study suggested that it depends on the length of the aliphatic chains and the presence of double bonds [72], highlighting the need for more research.

The ability to reduce oxidative stress through the scavenging of free radicals is highly relevant for the treatment of skin disorders [73]. The present study reveals that among the hydrolates and EOs examined, only *O. basilicum* EO exhibits strong antioxidant activity by effectively neutralizing the DPPH radical. This finding aligns with the activity observed by Mohammed et al. [74] and Hussain et al. [75]. However, studies have found opposing outcomes, indicating low antioxidant capacity, potentially associated with the origin of the plants. Also, the EO from *T. capitata* showed moderate activity in scavenging DPPH free radicals, which might be related to the high content of the phenol carvacrol, according to the authors [76]. Other studies addressing the *M. chamomilla* EO showed high antioxidant activity with an IC_50_ of 100 µg/mL [77], even though the authors found monoterpenes in the EOs’ composition, in contrast with the sesquiterpenes reported in this study.

The biocompatibility of hydrolates and EOs was assessed through various in vitro models of skin-related cells. The hydrolates at high concentrations were able to reduce the cell proliferation on the tested cell lines, with RAW cells being the most susceptible here. On the other hand, EOs presented significant decreases in cell viability starting from the lowest concentrations on the tested cell lines. When RAW 264.7 macrophages were exposed to *T. capitata* hydrolate and EO, a significant reduction in cell viability was observed only at an EO concentration of 256 µg/mL. In contrast, the hydrolate showed no effect on cell proliferation across all tested concentrations (25–400 µg/mL) [78]. In the same study, both the hydrolate and EO were also tested on 3T3 fibroblasts, where the EO induced a non-significant decrease in cell viability at concentrations above 128 µg/mL [78]. Comparing these results with the present study and accounting for a density below 1 g/mL, the EO concentration of 256 µg/mL reported by Alves-Silva, Pedreiro, Cavaleiro, Cruz, Figueirinha and Salgueiro [78] equates to 0.000256 g/mL. Dividing by the density yields a value lower than 0.0256% *v*/*v*. Therefore, a significantly higher concentration was tested in the present study, which accounts for the lower cell viability observed. The same procedure can be applied to the hydrolate. *O. basilicum* EO exhibited cytotoxic effects against NIH 3T3 fibroblasts, with an IC_50_ value of 120.7 ug/mL [79]. These findings contradict the present study; however, the discrepancy may be attributed to the use of different fibroblast cell lines. Specifically, NIH 3T3 cells are derived from mouse embryonic fibroblasts, whereas the cell line employed here (ATCC^®^ CRL-1634) consists of finite human fibroblasts isolated from newborn foreskin. To the author’s best knowledge, there is no available data in the literature about the cytotoxic evaluation of *M. chamomille* and *E. purpurea* hydrolates. The production of ROS is an indicator of the inflammation process, which involves NO as a signaling molecule [80]. Also, NO plays an important role in the control of skin-related processes such as the wound healing, proliferation and differentiation of epidermal cells, microbicidal activity and the regulation of innate immune reactions [80]. This study showed that all hydrolases, as well as the *T. capitata* EO, reduced the nitrite levels by scavenging NO present in the cells’ medium. This finding could suggest that they cannot interfere with the inflammation process. In contrast, the *O. basilicum* EO successfully inhibited the NO production in LPS-stimulated macrophages, suggesting a strong anti-inflammatory activity, which may be related to the expression of the iNOS protein [80]. Another study focused on the same EO identified a suppression of the COX-2 protein activity, a known inflammatory promoter, on human macrophage-like cells [81]. Yet, to the best of the author’s knowledge, no further data were accessible regarding the anti-inflammatory activity of hydrolates or EOs pertaining to the plants under study. Nevertheless, it is worth mentioning that some of the chemical compounds found in this study have previously shown such activity. For example, eugenol, identified as the major compound of *O. basilicum* hydrolate in another study of the group [49], was found to possess anti-inflammatory effects by inhibiting the expression of the NO synthase (iNOS) on RAW 264.7 cells [82]. Furthermore, carvacrol, found in *E. purpurea* hydrolate and *T. capitata* hydrolate and EO, previously exhibited inhibitory effects on the production of NO and other inflammation-promoting proteins [83], besides preventing cell death through a protective effect against pro-inflammatory activation on LPS-stimulated RAW 264.7 macrophages [83].

## 4. Materials and Methods

### 4.1. Plant Preparations

The *Matricaria chamomilla* flowers were purchased from the company “Distriplant” (Entroncamento, western Portugal), being harvested between September and October of 2019, and *Echinacea purpurea* aerial parts (flowers, stems and leaves) were purchased from the company “Ervas da Zoé” (Idanha-a-Nova, center Portugal), harvested between November and December 2019. *Thymbra capitata*, aerial parts (flowers, stems and leaves) harvested in May-June 2019, and the *Ocimum basilicum* leaves harvested in August 2018, were obtained directly from the aromatic and medicinal plant producers located in Proença-a-Nova (center Portugal) and Covilhã (center Portugal) respectively. The hydrolates and EOs were obtained (as final products) by hydrodistillation using a Clevenger-type apparatus as described by The European Pharmacopeia [84]. About 100 g of the dried flowering parts of *M. chamomilla*, 50 g of the dried aerial parts (flowers, stems, and leaves) of *E. purpurea* and *O. basilicum*, and 17.15 g of the dried aerial parts (flowers, stems, and leaves) of *T. capitata* were submerged in distilled water and subjected to hydrodistillation for 2 h. The obtained hydrolates and EOs were stored in dark vials at 4 °C.

### 4.2. Chemical Characterization

The plant hydrolates and EOs from *M. chamomilla* and *E. purpurea* were analyzed by gas chromatography-mass spectrometry (GC/MS SCION SQ 456 GC, Bruker, Billerica, MA, USA) according to the protocol described by Lisec et al. [85]. The chemical characterization of hydrolates and EOs of *Thymbra capitata* and *Ocimum basilicum*, is not described in this paper because they were already reported in a previous study from the group [49].

The separation of all compounds was performed through HP-5MS fused silica capillary column (Agilent J&W GC Columns, 30 m × 0.25 mm × 0.25 µm, Santa Clara, CA, USA) and helium X50S_BIP as a carrier gas with a flow rate of 1 mL/min. The hydrolates of *M. chamomilla* and *E. purpurea* were injected at a concentration of 6 mg/mL (1.2 µL) in a ratio of 1:5; 4 mg/mL (1.0 µL) in a ratio of 1:10; 1 mg/mL (2 µL) in a ratio of 1:5, respectively. The EO of *M. chamomilla* was previously diluted with pentane (1:500) and injected (0.2 µL) in a ratio of 1:50. The oven initial temperature was set at 45 °C with a gradual increase of 3 °C/min, after it had reached 175 °C the temperature was set to increase 15 °C/min until reached 300 °C and remained at this temperature for the final 10 min. The injector and detector temperatures were kept at 220 and 250 °C respectively. The identification of the volatile compounds was accomplished by three different analytical methods, the comparison of the mass spectrometric data obtained with the mass spectrometers from the W-Package MS-Library NIST 17 database included in the equipment’s software, the comparison of the mass spectrometric data with the Kovats retention index (NIST 17) and the comparison of these with the experimental Kovats index acquired from a series of alkanes injected according to the same methods as the sample. The remaining compounds were identified by comparison with standards. The compound’s relative amount was shown as a percentage by comparison between the relative area of the compound peak and the total area of the compound peaks identified in the sample, and as a percentage of the compound’s peak area relative to the area of the major compound peak.

### 4.3. Antimicrobial Activity

The antimicrobial activity was tested against skin microbiota (Gram-positive: *Staphylococcus aureus* (ATCC 6538) and *Cutibacterium acnes* (DSMZ 1897), and Gram-negative: *Escherichia coli* (ATCC 8739) and *Pseudomonas aeruginosa* (ATCC 9027) through the microdilution method in 96-well plates. Culture media Tryptic soy agar (TSA, Sigma-Aldrich, Burlington, MA, USA) was prepared in distilled water (Water Milli-Q) for aerobic bacteria and brain–heart infusion (BHI, Sigma-Aldrich, Burlington, MA, USA) supplemented with 15 g/L of agar and 5% glucose for anaerobic bacteria. The media were then autoclaved at a high temperature (121 °C, 15 min) for sterilization. The bacteria were undergoing growth within an incubator maintained at 37 °C. Additionally, the Anaerocult^®^ A (Sigma-Aldrich, Burlington, MA, USA) anaerobic system was employed to create an anaerobic environment specifically tailored for the growth of *Cutibacterium acnes*.

The assay was performed according to the Clinical and Laboratory Standards Institute M07-A10, M11-A8, M27-A3, and M38-A2 microdilution methods for testing aerobic and anaerobic bacteria, yeast and fungi [86,87,88,89]. In short, bacterial cultures were used to create a 0.5 MacFarland suspension by utilizing sterile 0.85% (*w*/*v*) NaCl. Subsequently, appropriate dilutions were made using various culture media. The plant preparations were subjected to serial dilution within 96-well plates prior to the addition of microbial suspensions. Hydrolates and EOs solutions were tested at various concentrations ranging from 0.78 to 50% and 0.004–0.25% (*v*/*v*), respectively. Also, to enable the dilution of EOs within the culture media, sterile DMSO was used as a surfactant. The adjusted microbial suspensions were subjected to serial dilutions of hydrolates and EOs, resulting in a 2-fold dilution for the plant preparations and the microbial suspensions.

The different controls used include microbial suspensions inoculated in culture media (positive control with maximum growth) and non-inoculated media (negative control without growth), but also DMSO at the maximum concentration to eliminate any doubtful interference from this surfactant. Moreover, a range of antibiotic agents with diverse spectrums of action were employed as positive controls for assessing antimicrobial activity, such as ampicillin (max. 16 μg/mL), erythromycin (max. 16 μg/mL), gentamicin (max. 4 μg/mL), tetracycline (max. 4 μg/mL), ciprofloxacin (max. 2 μg/mL) and benzylpenicillin (max. 1 μg/mL). At the end, the minimum inhibitory concentration (MIC), defined as the first concentration that caused a complete absence of microbial growth, was visually determined by 96-well plates examination.

### 4.4. Antioxidant Activity

The free radical-scavenging potential of both hydrolates and EOs was evaluated through the 2,2-Diphenyl-1-picrylhydrazyl [(DPPH)-Sigma Aldrich, Burlington, MA, USA] through the method proposed by Molyneux [90], but performed in a microplate as proposed by Prieto [91]. The DPPH solution was prepared in methanol (0.05 mg/mL) and 100 µL added to 100 µL of serially diluted hydrolates and EOs, yielding a 2-fold dilution. The plates were incubated for 30 min at room temperature. A negative control composed of DPPH stock solution mixed with methanol (0.025 mg/mL), and Ascorbic acid (Fisher Chemical, Waltham, MA, USA) was used as the positive control. The microplates were stored in the dark at room temperature for 30 min and then the absorbance was read at 517 nm. The DPPH radical-scavenging was calculated through the following Equation (1), as described by Rocha et al. [92]:

Reduction (%) = 100 ((Abs of sample − Abs of blank)/(Abs of control) × 100)(1)

“Abs sample” is the absorbance of DPPH in the presence of the sample. “Abs blank” is the basal absorbance of the sample in methanol. “Abs control” is the absorbance of the negative control. The IC_50_, which is the estimated concentration of an antioxidant necessary to cause a 50% inhibition of free radical activity, was determined using a calibration curve. This was obtained through a linear regression between the different concentrations and the corresponding percentage of DPPH reduction. To express the antioxidant activity, the antioxidant activity index (AAI) already described by Scherer et al. [93] was used:

AAI = (DPPH final concentration)/(Sample IC_50_) (2)

If AAI < 0.5, the antioxidant activity is poor; 0.5 < AAI < 1.0, the antioxidant activity is moderate; 1.0 < AAI < 2.0, the antioxidant activity is strong.

### 4.5. Cytotoxicity

The hydrolates and EOs cytotoxicity were verified in the cell lines of macrophages (RAW 264.7, ATCC^®^, TIB-71^TM^), fibroblasts (3T3, ATCC^®^ CRL1634^TM^), and keratinocytes (HaCaT; CLS 300493). The Dulbecco’s Modified Eagle’s Medium―DMEM (Gibco, Alfagene, Lisboa, Portugal), supplemented with 25 mM of D-Glucose (Sigma-Aldrich, Burlington, MA, USA) and 17.95 mM of sodium bicarbonate, was used to grow the RAW cells; and the DMEM (Gibco, Alfagene, Lisboa, Portugal) supplemented with 39.90 mM of sodium bicarbonate (Sigma-Aldrich) was used to grow the 3T3 and HaCaT cells. Also, 100 U/mL of penicillin and 100 Ug/mL of streptomycin (Sigma-Aldrich, Burlington, MA, USA), and 10% of fetal bovine serum (FBS, Sigma-Aldrich), which were inactivated for 3T3 and HaCaT, were added to each medium. The cell sub-culturing was performed according to ATCC recommendations at 37 °C in a 95% air and 5% CO_2_ humidified atmosphere. To detach the cells after reaching the desired confluence, a cell scraper (RAW) or a Trypsin-EDTA solution (3T3 and HaCaT) was used, and the number of viable cells was evaluated through the trypan-blue dye to perform the in vitro assays. The 3T3 and HaCaT cells were seeded at a density of 1 × 10^4^ in microplates with 96-wells, whereas the RAW cells were seeded at a density of 2.5 × 10^4^ cells per well. After 24 h of incubation in 95% air and 5% CO_2_ humidified atmosphere, the hydrolates and EOs solutions were prepared in culture medium using a series of concentrations ranging from 0.78 to 50% and 0.004–0.25% (*v*/*v*), respectively. These solutions were added to each well containing the cells during a 24 h period of incubation in 95% air and 5% CO_2_ humidified atmosphere.

Hydrolates were tested at the same concentrations as for antimicrobial activity, but the EOs were tested at the highest concentration in which the solvent, DMSO, did not cause a decrease in cell viability higher than 30%. The cytotoxicity was determined by assessing the 3-(4,5-dimethylthiazol-2-yl)-2,5-diphenyltetrazolium bromide (MTT) colorimetric method, adapted from reference [94]. After 24h of exposure, the hydrolates and EOs solutions were removed, and a PBS wash was performed in each well. Subsequently, 100 µL of MTT stock solution, prepared in incomplete medium with the final concentration of 5 mg/mL, was added to each well. The plates were then incubated at 37 °C for 4 h in a humidified incubator containing 5% CO_2_. Thereafter, isopropanol (Sigma-Aldrich, Burlington, MA, USA) was added to each well and thoroughly mixed to dissolve the dark blue formazan crystals. The formazan quantification was evaluated using the xMark^TM^ Microplate Absorbance Spectrophotometer (Bio-Rad, Hercules, CA, USA) at 570 nm, with a reference wavelength of 620 nm. The results were expressed as a percentage of metabolic activity in comparison to the control group.

### 4.6. Anti-Inflammatory Activity

#### 4.6.1. NO Production

For the analysis of NO production by macrophages (RAW cell line), the cells were seeded at a density of 1 × 10^5^ cells/mL in 96-well plates and exposed to the hydrolates and EOs solutions, diluted with DMEM medium, in the presence or absence of LPS (1 µg/mL), for 24 h. Then, the NO production was determined in supernatants using Griess colorimetric nitrite assay by mixing an equal volume of cell culture supernatant with an equal volume of Griess reagent (Reagent A prepared with Sulphanilamide 1% (*w*/*v*) (Sigma-Aldrich, Burlington, MA, USA) in phosphoric acid 5% (*v*/*v*)) (Sigma-Aldrich, Burlington, MA, USA) and Reagent B prepared with 0.1 g N-1-naphthylenediamine 0.1% (*w*/*v*)) (Sigma-Aldrich, Burlington, MA, USA). The plate was placed in the dark, at room temperature, for 30 min. The absorbance was measured at 550 nm, and nitrite concentration was calculated through a regression analysis of a sodium nitrite standard curve (NaNO_2_).

#### 4.6.2. Scavenging Activity

The evaluation of the scavenging potential was conducted following the protocol described by Roxo et al. [55]. The solution of S-Nitroso-N-acetylpenicillamine (SNAP, Sigma-Aldrich, Burlington, MA, USA) at 300 µM, prepared in DMSO, was added to each well, which had previously contained hydrolates and EOs solutions prepared with LPS-free DMEM. Wells containing only culture medium (without SNAP) were included and worked as a negative control. The plates were incubated for 3 h at 37 °C, in a 95% air and 5% CO_2_ humidified atmosphere. Afterwards, the Griess test was performed, and the absorbance was measured using a xMarkTM Spectrophotometer (Bio-Rad, Hercules, CA, USA) at a wavelength of 550 nm. The results were presented as a percentage of nitrite levels present in the medium with and without SNAP.

## 5. Conclusions

Hydrolates and EOs obtained through hydrodistillation are considered to contain the inherent bioactive compounds found in plants. These secondary metabolites, produced by plants, are recognized for their potential as agents possessing antimicrobial, antioxidant and anti-inflammatory properties. This study aimed to evaluate those aspects in order to provide valuable insights about the potential of these natural ingredients for application on the human skin. Upon analyzing the chemical characterization of the hydrolates and EOs, it was observed that both sesquiterpenes and monoterpenes were identified as the predominant compounds. Specifically, the *O. basilicum* EO exhibited high antimicrobial, antioxidant, and anti-inflammatory activities while also demonstrating low to moderate toxicity. This suggests a beneficial balance between the natural properties of *O. basilicum* EO and its biocompatibility, possibly aiding the skin healing process by decreasing the accumulation of reactive oxygen species responsible for the healing process. Ultimately, these attributes position *O. basilicum* EO as a promising option for therapeutic use, as an active ingredient in topical formulations for the treatment of skin diseases.

## Figures and Tables

**Table 1:** Chemical characterization of plant extracts determined by GC-MS analysis. The proportion of the predominant compound in each extract is expressed in percentage (concentration %), as well as its chemical class.

		*M. chamomilla* Hydrolate	*M. chamomilla* EO	*E. purpurea* Hydrolate	*E. purpurea* EO
Compound	RI ^a^	Peak Area ^b^ (%)			
**Alkanes**					
Pentacosane	51.44				0.54
**Aromatics** **/phenylpropanoids**					
2-Methoxy-4-vinylphenol Phenol, 5-ethenyl-2-	23.67			5.80	
Benzeneacetaldehyde	11.61			0.55	
Carvacrol	23.25			0.76	
Estragole	18.64				0.64
p-Cymen-8-ol	17.96			1.46	
methoxy-(2-Methoxy-4-vinylphenol) p-Mentha-1,4(8)-	24.31			1.24	
**Monoterpenes**					
1,5-Heptadien-4- one 3,3,6-trimethyl	12.61		0.59		
Bicyclo [3.1.1]heptan-3-ol, 6,6-dimethyl-2-methylene-, [1S-(1α,3α,5α)]-	15.79			1.94	
cis-Verbenol	15.95			1.37	
Citral	21.84			0.73	
D-Carvone	20.56			0.85	
endo-Borneol	17.03			0.59	
Geraniol	21.17			0.52	
Levoverbenone	19.00			9.49	
Linalool	14.19			0.97	
Myrtenal	18.47				0.60
Myrtenol	18.46			1.39	
dien-3-one (Piperitenone)	24.77			2.51	
p-Mentha-1,5-dien-8-ol	17.13			4.32	
p-Mentha-1,8-dien-7-ol	23.01			0.71	
Pulegone	20.35			1.04	
trans-Carveol	19.50			2.84	
trans-Verbenol	16.15c			11.70	
	16.19d				1.96
α-Campholenal	15.35				0.79
α-Citral	21.90				0.60
α-Terpineol	18.20			2.18	
β-Phellandrene	8.86	0.54			
**Sesquiterpenes**					
(-)-Spathulenol	34.42b		2.78		
	34.35c			0.70	
	34.43d				5.84
(1R, 7S, E)-7-Isopropyl-4,10-dimethylenecyclodec-5-enol	38.44				3.75
(E)-β-Farnesene	29.86		4.54		
1,5-Epoxysalvial-4(14)-ene	33.98				3.93
15-Hydroxy-alpha-muurolene	41.68				0.54
(Benzene, 1-(1,5-dimethyl-4-hexenyl)-4-methyl)	30.84				0.66
Bisabolol	38.45		0.56		
Bisabolol oxide A	40.60a	80.67			
	40.61b		62.42		
Bisabolol oxide B	37.34a	9.58			
	37.35b		10.50		
Bisabolone oxide A	38.34a	2.13			
	38.36b		7.99		
Caryophyllene	28.18				1.18
Caryophyllene oxide	34.62				9.52
Chamazulene	39.91		0.87		
cis-ene-yne-Dicycloether	44.67a	2.01			
	44.69b		6.52		
Cubebol	32.02				0.72
Epicubebol	31.22				0.66
Farnesyl acetone	45.42				0.64
Germacrene D	30.69				10.64
Humulene	29.53				0.58
Humulene epoxide II	34.62				5.61
Salvial-4(14)-en-1-one	35.02				2.35
Shyobunol	38.59				5.45
tau-Cadinol	36.82		0.94		
tau-Muurolol	36.86				1.56
β-Cadinene	32.40				1.07
**Fatty Acids**					
n-Decanoic acid	26.35		1.65		
n-Hexadecanoic acid	46.09				0.79
Alkanes		0.00	0.00	0.00	0.54
Aromatic/phenylpropanoids		0.00	0.00	9.81	0.64
Monoterpenes hydrocarbons		0.54	0.00	0.00	0.00
Oxygenated monoterpenes		0.00	0.59	43.15	3.95
Sesquiterpenes hydrocarbons		0.00	5.41	0.00	13.47
Oxygenated sesquiterpenes		94.39	91.71	0.7	41.23
Fatty Acids		0.00	1.65	0.00	0.79
Total Identified		94.93	99.36	53.66	60.62

^a^ RI: retention index calculated by standard n-alkane. ^b^ Peak area results represent compounds with percentages > 0.5% for both hydrolates and Eos; a, b, c and d correspond respectively to *M. chamomilla* hydrolate, *M. chamomilla* EO, *E. purpurea* hydrolate and *E. purpurea* EO.

**Table 2:** Antimicrobial activity (MICs) of plant hydrolates and EOs determined by microdilution broth assay. The values are expressed as % (*v*/*v*) and represent the value of at least two independent experiments.

Plant Extract	*E. coli*	*P. aeruginosa*	*S. aureus*	*C. albicans*	*C. acnes*	*A. brasiliensis*
*M. chamomilla* hydrolate	>50%	>50%	>50%	>50%	>50%	>50%
*E. purpurea* hydrolate	>50%	>50%	>50%	>50%	>50%	>50%
*T. capitata* hydrolate	25%	12.5%	12.5%	12.50%	25%	12.50%
*O. basilicum* hydrolate	>50%	>50%	>50%	>50%	>50%	>50%
*M. chamomilla* EO	a	a	a	a	a	a
*E. purpurea* EO	a	a	a	a	a	a
*T. capitata* EO	0.08%	0.08%	0.16%	0.0025%	0.03%	0.0025%
*O. basilicum* EO	1%	>2%	2%	>2%	0.06%	2%

^a^ Not possible to perform due to very low yield during the hydrodistillation process. MIC (Minimum Inhibitory Concentration) is determined as the lowest concentration of the tested compound that visibly inhibits microbial growth.

**Table 3:** Antioxidant capacity of hydrolate and EOs based on IAA and IC_50_.

	IC_50_	IAA	Antioxidant Capacity
Ascorbic acid	0.00026	9.61538	Very Strong
*M. chamomilla* hydrolate	47.81	0.00005	Low
*E. purpurea* hydrolate	32.90	0.00008	Low
*T. capitata* hydrolate	5.78	0.00043	Low
*O. basilicum* hydrolate	12.38	0.00020	Low
*M. chamomilla* EO	a	a	a
*E. purpurea* EO	a	a	a
*T. capitata* EO	0.012	0.208	Low
*O. basilicum* EO	0.002	1.272	Strong

^a^ Not possible to perform due to very low yield during the hydrodistillation process. IAA: index of antioxidant activity. IC_50_: extract concentration (%, *v*/*v*) able to reduce half of the DPPH molecules in test.

## Data Availability

The raw data can be obtained on request from the corresponding author.

## Abbreviations

The following abbreviations are used in this manuscript:

AAI	Antioxidant activity index
COX-2	Cyclooxygenase-2
DMEM	Dulbecco’s Modified Eagle’s Medium
DMSO	Dimethyl sulfoxide
DPPH	2,2-Diphenyl-1-picrylhydrazyl
EDTA	Ethylenediaminetetraacetic acid
EO	Essential oil
GC-MS	Gas chromatography-mass spectrometry
iNOS	Nitric oxide synthase
LPS	Lipopolysaccharide
MIC	Minimum inhibitory concentration
MTT	3-(4,5-dimethylthiazol-2-yl)-2,5-diphenyltetrazolium bromide
NO	Nitric oxide
PBS	Phosphate-buffered saline
ROS	Reactive oxygen species
SDS	Sodium dodecyl sulfate
SNAP	(3*S*)-1-[2-[tris(4-methoxyphenyl)methoxy]ethyl]piperidine-3-carboxylic acid
